# Altered fMRI Connectivity Dynamics in Temporal Lobe Epilepsy Might Explain Seizure Semiology

**DOI:** 10.3389/fneur.2014.00175

**Published:** 2014-09-11

**Authors:** Helmut Laufs, Roman Rodionov, Rachel Thornton, John Sydney Duncan, Louis Lemieux, Enzo Tagliazucchi

**Affiliations:** ^1^Department of Neurology and Brain Imaging Center, Goethe-University Frankfurt am Main, Frankfurt am Main, Germany; ^2^Department of Neurology, University Hospital Schleswig-Holstein, Christian-Albrechts-University Kiel, Kiel, Germany; ^3^National Hospital for Neurology and Neurosurgery, University College London, London, UK; ^4^The Epilepsy Society, Chalfont St. Peter, UK

**Keywords:** functional connectivity, non-stationarity, temporal lobe epilepsy, biomarker, EEG-fMRI, interictal epileptiform discharges, semiology, seizure

## Abstract

Temporal lobe epilepsy (TLE) can be conceptualized as a network disease. The network can be characterized by inter-regional functional connectivity, i.e., blood oxygen level-dependent (BOLD) signal correlations between any two regions. However, functional connectivity is not constant over time, thus computing correlation at a given time and then at some later time could give different results (non-stationarity). We hypothesized (1) that non-stationarities can be induced by epilepsy (e.g., interictal epileptic activity) increasing local signal variance and that (2) these transient events contribute to fluctuations in connectivity leading to pathological functioning, i.e., TLE semiology. We analyzed fMRI data from 27 patients with TLE and 22 healthy controls focusing on EEG-confirmed wake epochs only to protect against sleep-induced connectivity changes. Testing hypothesis (1), we identified brain regions where the BOLD signal variance was significantly greater in TLE than in controls: the temporal pole – including the hippocampus. Taking the latter as the seed region and testing hypothesis (2), we calculated the time-varying inter-regional correlation values (dynamic functional connectivity) to other brain regions and found greater connectivity variance in the TLE than the control group mainly in the precuneus, the supplementary and sensorimotor, and the frontal cortices. We conclude that the highest BOLD signal variance in the hippocampi is highly suggestive of a specific epilepsy-related effect. The altered connectivity dynamics in TLE patients might help to explain the hallmark semiological features of dyscognitive seizures including impaired consciousness (precuneus, frontal cortex), sensory disturbance, and motor automatisms (sensorimotor cortices, supplementary motor cortex). Accounting for the non-stationarity and state-dependence of functional connectivity are a prerequisite in the search for potential connectivity-derived biomarkers in TLE.

## Introduction

Epilepsy affects the brain both during seizures and interictally in the form of neurobehavioral problems. These are considered to be due to progressive structural and functional changes in the brain related to syndrome-specific “network” variations (1). In temporal lobe epilepsy (TLE), for example, interictal language and memory impairment are typical (2). In terms of seizure semiology, motor automatisms and consciousness impairment are characteristics of dyscognitive (complex partial) seizures typical for TLE. We hypothesized that, during seizure-free wakeful rest, brain activity is altered in syndrome-specific regions owing to so-called interictal epileptic activity – which may or may not be visible on scalp EEG (3) – and additionally any other sort of paroxysmal activity patterns differing from activity within a healthy brain, an “epileptic process.” Hence, as a first step, we tested the basic assumption that in TLE patients any such paroxysmal process should induce bursts of the blood oxygen level-dependent (BOLD) signal leading to increased regional variance compared to healthy controls. Among the primary candidate regions, we expected either one or both of those contributing to “interictal” cognitive impairment and those functionally related to temporal lobe seizure semiology, i.e., not only temporal (e.g., hippocampal) but also extratemporal (consciousness subserving and motor) regions.

Temporal lobe epilepsy can be considered as a network disease (4, 5) because widespread anatomic abnormality exists outside the primary epileptic zone affecting inter-regional connectivity linked to distributed cognitive impairments (6). Hence, a natural second step is to explore to which other brain regions – exhibiting “normal” signal fluctuations – the locations of increased variance are more tightly connected in the epileptic condition than in the healthy control subjects. By always taking healthy brain activity as the reference, we ensured that the observed typical effects were specific for the epileptic condition, in particular TLE.

However, it has been shown that functional connectivity is not static (7–11) but differs between different stages of wakefulness (12), and in addition fluctuates within each stage over time. “Dynamic functional connectivity” is here understood in the context of fluctuating, non-constant coupling between brain areas when the coupling is computed over short temporal windows. In contrast with the traditional practice of assuming a steady correlation between regions, the study of dynamic connectivity aims to study how connectivity evolves in time, how stable connections are and what the possible short-term connectivity motifs are between regions. Assuming a paroxysmal pathological process influencing brain activity in TLE, it is conceivable that accounting for the non-stationarity of inter-regional BOLD signal correlations both between and within wake and other sleep stages is mandatory for obtaining specific results with high sensitivity. Because of this, we selected epochs of data recorded during epochs of wakefulness and within these based all inferences on dynamic functional connectivity measures as obtained by means of sliding window analysis. Then, the variability of dynamical functional connectivity was computed (as the variance of the functional connectivity time series), which represents the intensity of the temporal fluctuations in the connectivity of the regions. A low value of variance of the dynamic connectivity time series corresponds to stable connections, in which coupling does not deviate much from the average value. In contrast, large values of the variance correspond to widely fluctuating connectivity, i.e., switching over time between high and low values of the coupling between regions.

## Materials and Methods

All procedures were subject to the relevant local and national research ethics committees’ approval.

### Subjects and patients

Data from 22 healthy subjects (11 females) served as control in this study (age 35 ± 12 years), and data from 27 epilepsy patients [13 classified as left TLE (age 32 ± 9 years), 14 with right (age 33 ± 11 years); 16 females], who were selected from a larger dataset with the inclusion criteria of TLE based on electro-clinical information-informed expert classification (Table 1).

**Table 1:** **Patient characteristics**.

	Age (years)	Ictal	Interictal	Structural MRI	Seizure semiology included		VIQ	PIQ	Epilepsy duration (years)	Medication
								
		EEG changes			Reduced conscious- ness	Motor automa- tisms	
L1	49	Left	Left > right	Normal	Yes	Yes	84	99	Unknown	Unretrievable
L2	29	Initial left	Left	Normal	Yes	No	100	100	14	LTG
L3	28	Left (icEEG)	Left (icEEG)	Normal	Yes	Yes	87	100	18	LEV, VPA
L4	33	Left	Left	Left HS	Yes	Yes	71	81	28	Unretrievable
L5	27	Left	Left	Left HS	Yes	Yes	90	99	24	LEV, VPA, PGB
L6	30	Left (icEEG)	Left (icEEG)	Signal change in calcarine fissure	Yes	Yes	75	80	15	CBZ, LEV, CZ
L7	41	Left	Left	Left HS	Yes	Yes	80	93	Unknown	CBZ, LEV
L8	48	Left	Left > right	Normal	Yes	Yes	107	119	37	LEV, PHT
L9	19	Left	Left	Left HS	Uncertain	Yes	92	98	12	TPM, LTG
L10	26	Left	Left	Bilateral HS	Yes	Yes	99	106	19	LEV, LTG
L11	42	Left	Left	Left HS and parietal WML	Yes	Yes	97	99	33	LEV, PHT, PGB
L12	28	Left	Left (icEEG)	Left STG abnormality	Yes	Negative motor	102	106	Unknown	CBZ, LEV, LTG
L13	19	Left	Left, bilateral	Irregularly lobulated mass left temporal into frontal lobe	Yes	Yes	111	106	2	LTG
R1	25	Right	Right	Tumor right temporal lobe	Uncertain	Never observed	105	100	6	CBZ, LTG
R2	38	Bilateral	Right	Right HS	Yes	Yes	103	96	Unknown	CBZ, TPM
R3	20	Unretrievable	Unretrievable	Right HS	Uncertain	Never observed	80	73	4	CBZ, LEV
R4	23	No scalp discharges	Right	Right temporal gliosis	Yes	Yes	80	84	23	OXC, CZM, ACM
R5	25	Right (icEEG)	Right and independent left	Right HS, right temporal DNET	Yes	Yes	Unknown	Unknown	Unretrievable	Unretrievable
R6	40	Right temporal and left fronto -central	Right » left	Right HS	Yes	Yes	Unknown	Unknown	Unretrievable	TPM, PHB, PGB
R7	59	Right	Right	Bilateral WML	Uncertain	Yes	87	89	54	LEV
R8	30	Right	Right	Normal	Yes	Yes	80	80	13	OXC, GBP, CZM
R9	18	Right	Right	Right temporal DNET	Yes	Yes	101	110	11	OXC
R10	37	Right	Right	Normal	Yes	Yes	67	92	14	CBZ, LTG
R11	24	Right	Right > left	Normal	Yes	Yes	82	105	5	VPA, PGB, CZM
R12	41	Right	Right	Right HS	No	No	108	110	22	CBZ, LEV
R13	37	No scalp discharges	Right	Right HS	Yes	No	85	81	14	OXC, ZNS
R14	43	Right	Right	Normal	Yes	No	115	113	26	LTG, LCS

*L[index], syndrome classified as left TLE, R[index], syndrome classified as right TLE; VIQ/PIQ, verbal/performance IQ (Wechsler D. WAIS III Administration and Scoring Manual, New York Psychological Corporation 1997); icEEG, intracranial EEG; HS, hippocampal sclerosis, DNET, dysembryoplastic neuroepithelial tumor; WML, white matter lesion(s); STG, superior temporal gyrus; ACT, acetazolamide; CBZ, carbamazepine; CZM, clobazam; LCS, lacosamide; LEV, levetiracetam; LTG, lamotrigine; OXC, oxcarbazepine; PGB, pregabalin; PHT, phenytoin; TPM, topiramate; VPA, valproate; ZNS, zonisamide*.

### EEG-fMRI acquisition

In this study, we assumed the presence of an “epileptic process” possibly reaching beyond epochs of interictal epileptic activity visible on scalp EEG. Accordingly, the EEG was not used for the detection of epileptiform EEG activity but to identify fMRI epochs of wakefulness in order to increase the sensitivity and specificity of our results (12).

EEG via a cap (modified Brain Cap MR, Easycap, Herrsching, Germany) was recorded continuously (sampling rate 5 kHz, low pass filter 1 kHz) during fMRI acquisition with an MRI-compatible EEG system (BrainAmp MR^+^ and Brain Vision Analyzer; Brain Products GmbH, Gilching, Germany) yielding two 20-min sessions consisting of 404 T2*-weighted single-shot gradient-echo echo-planar images (EPIs; echo time/repetition time, 30/3000 ms; flip angle, 90°; 43 2.5 mm interleaved slices; FOV, 24 cm × 24 cm; matrix 64 × 64) acquired continuously on a 3T Signa Excite HDX MRI scanner (General Electric, Milwaukee, WI, USA).

### EEG data preprocessing

MRI and pulse artifact correction were performed based on the average artifact subtraction (AAS) method (13, 14) as implemented in Vision Analyzer 2 (BrainProducts, Germany) resulting in EEG with a sampling rate of 250 Hz. EEG was re-referenced to common average. Sleep stages were scored manually by an expert according to the AASM criteria (15). Epochs other than wakefulness were erased from the analysis (by excluding the corresponding BOLD time courses from both the variance and dynamic functional connectivity analyses). Epochs of wakefulness shorter than 2 min were not included in the analysis.

### fMRI data preprocessing

Using Statistical Parametric Mapping (SPM 8, http://www.fil.ion.ucl.ac.uk/spm), EPI data were realigned, normalized (MNI space), and spatially smoothed (Gaussian kernel, 5 mm^3^ full width at half maximum). The time course of the average signal at the ventricles (CSF as given by FSL’s Ventricle Mask standard image) as well as motion-induced noise was regressed out. We did not remove the global brain signal to avoid the issue of induced anti-correlations (16). Also, since there are direct electrophysiological correlates of the global resting-state signal (17), we believe that its removal is arbitrary and results in a loss of information. fMRI data was band pass filtered in the range 0.01–0.1 Hz using a sixth order Butterworth filter as described previously (8). In all cases, both 20 min fMRI sessions were analyzed and then the results were averaged within every subject (to guarantee the assumption of statistical independence) prior to statistical testing.

Subjects displayed similar degrees of head displacement across groups. Controls: 0.06 ± 0.02 mm, Left TLE: 0.08 ± 0.04 mm, Right TLE: 0.08 ± 0.05 mm. No significant differences (*p* > 0.05) between groups were found. As an additional control, we reproduced the results after erasing volumes associated with high ( >0.3) head displacement amplitude (see Appendix).

### Data analysis

#### Computation of temporal variability

**Figure 1:**
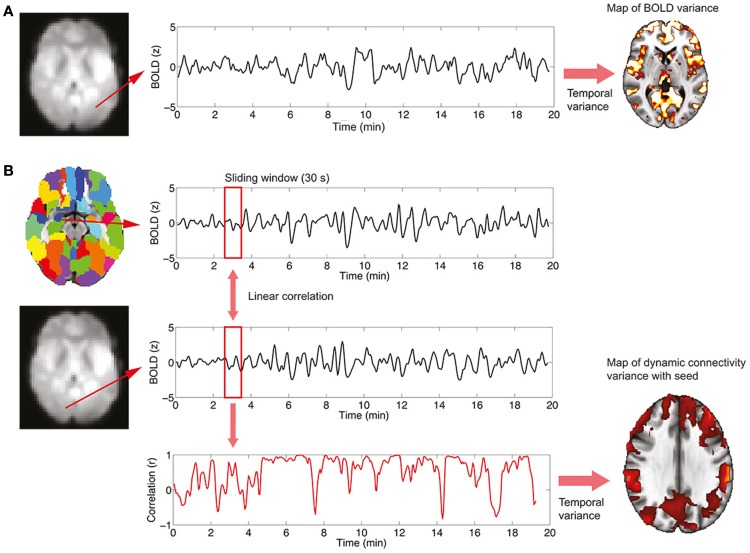
**Procedure to study BOLD signal and functional connectivity temporal variability**. **(A)** To compute the voxel-wise map of BOLD signal variability, the time series for every voxel in the EPI data is first extracted. Then, the variance of the signal is computed, resulting in the required map. **(B)** To compute the voxel-wise map of BOLD functional connectivity temporal variability with a seed, a region in the AAL template is first selected (in this work, the left and right hippocampi are used as seeds) and the average signal in the region is computed. Then, for every voxel in the EPI data, correlations over time are obtained using a sliding window (30 s) and the temporal dynamics of functional connectivity are computed. Note that in this example the dynamics are non-constant with moments of drastic loss of connectivity between regions. Finally, the temporal variance of the functional connectivity time series is computed, resulting in a spatial map (see Figure 4) encoding the variability in the interaction between the seed region and every voxel.

The temporal variability (variance) of BOLD signals carries important information about the brain state and also about task performance. An optimal value is typically observed during rest, which is decreased locally during task performance and increased in other brain states such as sleep (11, 18, 19). The possibility of interictal spikes occurring in TLE patient leads naturally to the hypothesis of locally increased variability (i.e., increased variance) of BOLD activity time courses. To quantify precisely the changes in variability observed in the patient vs. the control population, we use the traditional definition of variance in the time domain:

(1) $$\sigma^{2}=<{\left(X-<X>\right)}^{2}>$$

where X is the BOLD signal, σ^2^ the variance, and <, > denotes temporal averaging. This analysis is performed on a voxel-wise basis, resulting in spatial maps in which every voxel contains the variance of its associated time series. The procedure is summarized in Figure 1A.

#### Computation of functional connectivity variability

The study of functional connectivity between brain regions usually neglects the possibility of changes of connectivity occurring over time [*dynamical functional connectivity* (8)]. Not only functional connectivity values computed over extended periods of time characterize the healthy, resting brain but also a normal switching of connectivity values, representing the engagement and disengagement of different brain networks. At a first level, the normal level of this fluctuation in connectivity can be computed in two steps, which are also summarized graphically in Figure 1B:

(1) A temporal time course of functional connectivity is computed by means of a sliding window analysis. Inside each window, the normal correlation coefficient between the signal at a seed region and the signal at every voxel in the brain is computed. Then, the window is displaced one time step and the analysis repeated, obtaining one connectivity estimate per time unit. In this work, the sliding window length was set to 30 s, and the seed region to the left and right hippocampus [as defined in the automated anatomical labeling atlas (20)].
(2) Once the temporal time courses of functional connectivity are obtained, they are collapsed into a single spatial map by computing their temporal variability or variance (using Eq. 1). Thus, this results in a map in which every voxel has a value encoding how widely over time its connectivity with a seed region of interest is fluctuating.

### Statistical analysis

Both maps of temporal variability and of dynamical functional connectivity variability are compared between groups by means of mass univariate *t*-tests as implemented in the SPM8 software. All maps are reported at a level of *p* < 0.001 uncorrected with only clusters passing a threshold of *p* < 0.05 FWE corrected being shown.

## Results

**Figure 2:**
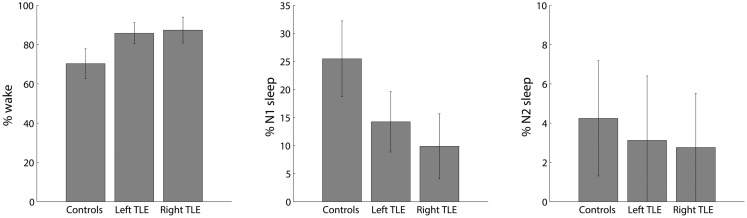
**Distribution of percent time spent in different sleep stages (wake, N1 and N2; no N3 sleep was observed) for healthy controls, left and right TLE patients (mean ± SEM)**.

On average, healthy controls slept more than left and right TLE patients (Figure 2). Controls were awake 71 ± 11% of the time, left TLE patients 85 ± 8%, and the right TLE patients 86 ± 9% of the time. This justifies the detection and deleting of BOLD data corresponding to sleep, considering the possibility of confounds due to comparing groups of subjects in different vigilance states (12).

**Figure 3:**
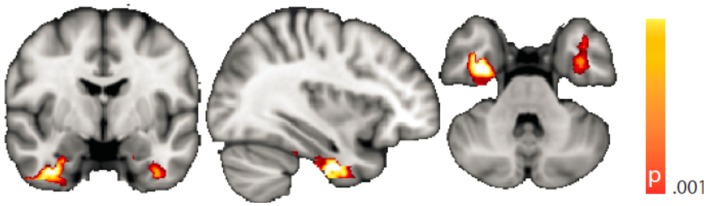
**Spatial map (coronal, sagittal, axial slices) of significantly greater variance of the blood oxygen level-dependent signal in patients with temporal lobe epilepsy (pooled right and left) than in healthy controls**. Color bar indicates *p*-value (thresholded at *p* < 0.001 for display, cluster survives family-wise error correction at *p* < 0.05). Left on figure is right in the brain (coronal and axial slices).

The voxel-wise analysis of BOLD signal variance in TLE was greater than in controls in the anterior temporal lobe bilaterally (Table 2; Figure 3). Significantly reduced variance compared to controls was not found in any voxel. A significant difference between left TLE and right TLE was not observed.

**Table 2:** **Regions, hemisphere, and statistical significance of areas with increased BOLD signal variance in TLE patients compared to healthy controls (local maxima at least 8 mm apart)**.

Brain region	MNI coordinates (*x*, *y*, *z*)	Hemisphere	*t*-Value
**Left + Right TLE > Healthy Controls**			
Inferior temporal gyrus	(38, −4, −36)	Right	6.44
Temporal pole	(−38, 8, −30)	Left	5.30
**Left TLE > Healthy Controls**			
Temporal pole	(36, 0, −36)	Right	5.31
**Right TLE > Healthy Controls**			
Fusiform gyrus	(34, −8, −36)	Right	5.08
Inferior temporal gyrus	(−36, −4, −36)	Left	4.89

*TLE, temporal lobe epilepsy*.

**Figure 4:**
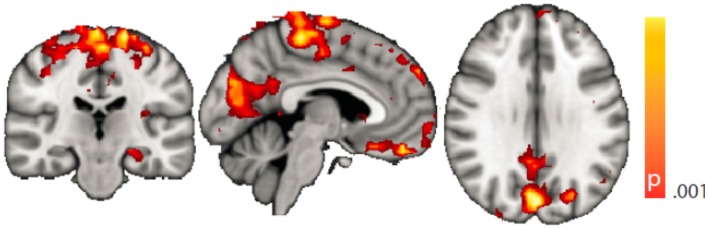
**Spatial map (coronal, sagittal, axial slices) of significantly greater variance of hippocampal dynamic functional connectivity in patients with temporal lobe epilepsy (here: seed in left hippocampus) than in healthy controls**. For differences between seeding in the left hippocampus and in the right, see Tables 3 and 4, of the pooled analysis, only results for the left hippocampus as the seed region are displayed (seed in right hippocampus yielded most similar results). Color bar indicates *p*-value (thresholded at *p* < 0.001 for display, cluster survives family-wise error correction at *p* < 0.05). Left on figure is right in the brain (coronal and axial slices).

Dynamic functional connectivity from the hippocampi in TLE (left and right TLE pooled) was greater than in controls in the supplementary motor area, the pre- and postcentral gyri, the (pre-)cuneus, the calcarine/middle occipital gyrus, and the superior frontal gyrus (Tables 3 and 4; Figure 4). A significant difference between left TLE and right TLE was not observed (compare Tables 3 and 4 for seeding in the left and right hippocampus, respectively). Of the pooled analysis, only results for the left hippocampus as the seed region are displayed and differences between seeding in the left and the right hippocampus were not significant (compare Tables 3 and 4).

**Table 3:** **Regions, hemisphere, and statistical significance of areas with increased variance of dynamic functional connectivity with the left hippocampus in patients compared to healthy controls**.

Brain region	MNI coordinates (*x*, *y*, *z*)	Hemisphere	*t*-Value
**Left + Right TLE > Healthy Controls**			
Supp. motor area	(4, −12, 72)	Right	4.78
Postcentral gyrus	(−20, −24, 72)	Left	4.95
Precentral gyrus	(−44, 4, 52)	Left	4.81
Cuneus	(1, −76, 28)	Right	4.97
Calcarine	(1, −92, −8)	Right	4.66
Sup. frontal gyrus	(1, 76, 8)	Right	4.79
Middle frontal gyrus	(−12, 79, 24)	Left	3.74
**Left TLE > Healthy Controls**			
Paracentral lobule	(0, −28, 80)	Left/right	4.76
Postcentral gyrus	(36, −28, 60)	Right	4.56
Cuneus	(12, −76, 20)	Right	5.14
Sup. frontal gyrus	(−8, 24, −20)	Left	4.56
Parahippocampal gyrus	(−32, −24, −20)	Left	4.51
Middle frontal gyrus	(−12, 75, 22)	Left	3.64
**Right TLE > Healthy Controls**			
Supp. motor area	(0, −20, 64)	Left/right	4.57
Cuneus	(0, −80, 28)	Left/right	4.95
Middle cingulate gyrus	(−1, −24, 52)	Left	4.31
Sup. frontal gyrus	(0, 76, 8)	Left/right	4.33
Parahippocampal gyrus	(−32, −24, −21)	Left	4.15
Middle frontal gyrus	(−7, 83, 32)	Left	4.01

*TLE, temporal lobe epilepsy, supp, supplementary, sup, superior*.

**Table 4:** **Regions, hemisphere, and statistical significance of areas with increased variance of dynamic functional connectivity with the right hippocampus in patients compared to healthy controls**.

Brain region	MNI coordinates (*x*, *y*, *z*)	Hemisphere	*t*-Value
**Left + Right TLE > Healthy Controls**			
Paracentral lobule	(0, −28, 80)	Left/right	5.10
Precuneus	(−8, −36, 60)	Left	4.35
Cuneus	(−12, −84, 40)	Left	4.82
Middle occipital gyrus	(28, −84, 28)	Right	4.84
Postcentral gyrus	(8, −40, 80)	Right	4.35
Sup. frontal gyrus	(0, 56, −20)	Left/right	4.12
**Left TLE > Healthy Controls**			
Paracentral lobule	(0, −24, 76)	Left/right	4.46
Precentral gyrus	(−40, −16, 64)	Left	4.06
Cuneus	(12, −96, 16)	Right	4.42
Fusiform gyrus	(−28, −24, −24)	Left	4.10
Middle frontal gyrus	(4, 84, −16)	Right	4.19
**Right TLE > Healthy Controls**			
Paracentral lobule	(0, −28, 80)	Left/right	4.26
Cuneus	(4, −80, 24)	Right	4.74
Middle frontal gyrus	(0, 60, 28)	Left/right	4.04
Inferior frontal gyrus	(48, 32, −16)	Right	4.15
Middle cingulate gyrus	(4, 36, 32)	Right	4.09

*sup, superior*.

## Discussion

Interictally, compared to healthy controls and during wakefulness, in the TLE group we found increased BOLD signal variance in the anterior temporal lobe regions overlapping with the left and right hippocampi and that these regions showed increased dynamic functional connectivity most prominently to sensory motor structures, the precuneus, and superior frontal cortices.

The identification of anterior temporal lobe structures in particular including the hippocampi is highly suggestive of a syndrome-specific effect because these regions have long been known as crucial in TLE (21). The anterior hippocampus is thought to be highly epileptogenic (22). In the context of interictal neuropsychological dysfunction, it is essential for memory processes (23), and with respect to seizure control, its surgical removal has been shown to be associated with a good outcome (24). In addition, our study design in which we try to keep constant as many parameters as possible between the control and the patient group (including the state of wakefulness) ensures that any significant group differences are specific to the condition TLE. In particular, we can identify significant activity alterations in sets of brain regions constituting intrinsic connectivity networks, which can be present both in the patient and the control group and which might evade other analysis techniques such as independent component analysis or conventional seed region-based functional connectivity analysis.

### Interpretation of increased hippocampal BOLD signal variance

The first observation that in our group of TLE patients, we found the highest BOLD signal variance in the anterior temporal lobe including, of note, the hippocampus supports our hypothesis of the presence of paroxysmal interictal activity resulting in regionally increased BOLD signal variance. It is well-established that local field potentials (LFP) explain the largest portion of this variance (25) and that interictal epileptic spikes in the anterior temporal lobe are reflected in LFP changes (26). We hence propose interictal epileptic activity (“epileptic process”) as the most likely explanation for the high anterior temporal lobe variance common to our TLE group. Kobayashi and colleagues as well as ourselves previously identified hippocampal BOLD signal increases at the group level in TLE, when correlating by means of a general linear model (GLM) interictal scalp EEG activity with the BOLD signal (27, 28). For these GLM-based EEG-fMRI studies, the occurrence of a number of interictal spikes was essential, whereas in the present approach, we on purpose designed our analysis to be independent of scalp EEG interictal activity. We did this for two reasons: (1) the sensitivity of scalp EEG in TLE is compromised with respect to hippocampal interictal epileptic activity (3, 29), allowing the creation of a scalp EEG-based GLM with limited sensitivity only and (2) we sought to identify an fMRI marker for paroxysmal activity specific to the epileptic condition independently of the simultaneously recorded EEG. Such paroxysmal activity in addition to “true” interictal spikes (as can be demonstrated with intracranial EEG) might include other types of epileptic (neuronal) activity leading to bursts of energy consuming processes, e.g., owing to damaged cells in the hippocampus, alterations in the glial milieu, or pathology of blood flow parameters (30). The exclusive selection of epochs during which the patients and control subjects, respectively, were awake ensured increased sensitivity when comparing resting-state BOLD signal properties: it is well known that the functional architecture of the brain changes significantly depending on the level of wakefulness (12, 31) including the variance of the BOLD signal in different brain regions (11, 32), but also graph theoretical network measures (33, 34), and variance in the brain’s functional connectivity structure (8).

We note that a very similar result (increased variance of BOLD signals in bilateral hippocampi) was recently found for subjects under the influence of a psychedelic substance (psilocybin) (35). Furthermore, a related result is discussed in the context of REM sleep (36), and in the psilocybin study the intensity of the “dream-like” experience, which was rated subjectively by the subjects, correlated positively with the magnitude of the hippocampal BOLD signal fluctuations. It can be speculated that subjective alterations in the conscious awareness of the patients – more subtle than full-blown seizures, but which can escalate into the former and also into auras – are shared in TLE and in the other altered states of consciousness mentioned above.

### Interpretation of increased hippocampal dynamic functional connectivity

Increased variance of dynamic functional connectivity in TLE compared to healthy controls indicates region pairs, which exhibit stronger fluctuations of the correlation of their BOLD time series in the epileptic condition than in the healthy. While the measure does not assess an absolute difference in connectivity between the two cohorts, regions are identified, which link and unlink typically more in TLE than in the control group. Our study design motivates the hypothesis that the “epileptic process” is responsible for this coupling and uncoupling, possibly in the form of the interictal epileptiform activity in the hippocampus, which leads to increased functional connectivity variance. Further analyses should reveal whether such epileptic activity weakens or strengthens the functional connection between regions. It may even be that “erratic” hippocampal coupling (and uncoupling) with cortical regions disrupts the physiological interplay of the latter with yet further parts of the brain – including subcortical and that this eventually results in the observed dysfunctionality.

Van Paesschen and colleagues used single photon emission computed tomography (SPECT) to study patients with hippocampal sclerosis (HS) and observed both ictal hyper- and hypoperfusion in (lateralized) regions partly overlapping with those we describe here in our study, which also includes many cases of HS. Hyperperfusion was described in temporal lobe, middle frontal and central regions, and in the frontal lobes and the precuneus hypoperfusion was found (37). It hence appears that both a “gain” as well as a “loss of function” in different brain regions is linked with ictal dysfunctioning. For example, Chassagnon and colleagues studied patients with mesial TLE and found ictal–interictal hypoperfusion in the posterior cingulate and prefrontal regions, which might be interpreted as a loss of function in the sense of impaired consciousness (precuneus) and executive functioning (frontal regions) (38).

Looking at the particular brain regions in TLE with increased connectivity variance, we speculate that (1) the hippocampal activity interferes with language (39) and memory function (40), both interictally and ictally and that (2) the increased dynamic connectivity to the precuneus and frontal cortex is ictally associated with impaired consciousness (4, 41) and executive functioning (42). We also propose that (3) the increased dynamics in functional connectivity between the hippocampus and the sensorimotor cortices might pave the way for ictal sensory and motor dysfunction and – probably tightly related to the supplementary motor area – in particular motor automatisms (43). The superior frontal gyrus was also described relevant for introspection (44).

### How are interictal connectivity changes linked with ictal semiology?

Our study does not offer any objective clues as to what the changes we describe in the TLE group interictally have to do with ictal changes in brain function. While it is well described that language and memory impairment are present interictally (45), sensorimotor dysfunction does not obviously occur interictally. One explanation could be that a qualitative difference between what is called interictal and ictal activity may not exist as such, but rather a quantitative one: Binnie a good decade ago pointed out that if only we tested carefully enough, transitory cognitive impairment could be related to “interictal” activity in many individuals (46). In our own EEG-fMRI study looking at BOLD signal changes related to formally interictal activity, we interpreted signal changes in regions of the so-called default mode network to explain reduced consciousness during dyscognitive seizures. However, we selected for the study cohort patients with very frequent interictal discharges on the EEG increasing the sensitivity of our discharge-correlated GLM analysis (27). With this in mind and taking Binnie’s idea forward, it is conceivable that with so-called interictal activity vastly the same set of brain regions (network) is recruited as is ictally with the difference that when behavioral alterations become obvious they are called seizures and hence define “ictal activity.” Of course, some additional features distinguish seizure from interictal activity going beyond “duration” alone but include spreading of epileptic activity. Still, such spreading of activity might occur along interictally pre-existing paths (47). Supporting our speculation further, in TLE, structural changes have been shown to progress over time and memory function was more closely related to structural hippocampal changes than the overt seizure frequency: the group of Bernasconi found neocortical thinning in TLE progressing over time in bilateral frontal (sensorimotor) and temporal (hippocampal, entorhinal, temporo-polar) regions – overlapping with the regions we report here (48), and Pacagnella and colleagues most recently presented data proposing that memory impairment is more influenced by hippocampal damage than by seizure frequency (40). In our limited cohort, we failed to identify a correlation between epilepsy duration and BOLD signal variance or dynamic functional connectivity (analysis not shown).

### Unidirectional differences and lack of significant lateralization of our findings

We did not find any significantly lateralized or side-dependent results. Instead, significant differences between healthy subjects and controls were usually bilateral. This might be due to a lack of sensitivity of our analysis, which was not optimized for this purpose (balancing of handedness, type of left- and right-sided pathologies, and EEG abnormalities). We hence do not discuss our lateralized results. In general, however, on first sight, a lack of lateralized findings is surprising taking into account clinical practice and surgical success with unilateral resections and the efforts spent with non- and invasive video-EEG telemetry and imaging to identify in which temporal lobe (hippocampus) the epileptogenic zone is located (49). Although it is clinically not a contested issue that it is relevant indeed to operate on the correct side of the brain, we are not aware of any systematic review of epilepsy surgery cases in which – for whatever reason – retrospectively the wrong side was operated upon. It is likely that the vastly symmetrical results we present reflect secondary bilateral “network” effects of a lateralized primary cause.

It is equally interesting that we found exclusively variance increases – and not any decreases – of BOLD signal amplitude and hippocampal functional connectivity in patients with TLE compared to control subjects. However, we would like to note that this does not rule out the possibility of decreased absolute functional inter-regional connectivity in the epilepsy cohort, as this differs from the variance of functional connectivity, which we report in our study and the interpretation of which is discussed above. Still, a relationship between the two measures might exist because many of the regions in which we found altered dynamic functional connectivity were also reported by Haneef and colleagues. They compared TLE patients to controls and found that the classical measure of static hippocampal functional connectivity was greater to the bilateral temporal lobes, insula, fornix, frontal poles, angular gyrus, basal ganglia, thalamus, and cerebellum. They found reduced connectivity with the occipital pole, calcarine, lingual, precuneus, sensorimotor cortex, and parts of insula and frontal lobes as well as medial frontal areas (50). We report results of a “Standard Seed Correlation Analysis” of our data in the Appendix. Because we analyzed wakefulness epochs exclusively, comparability to other data remains limited.

### Limitations

**Figure 5:**
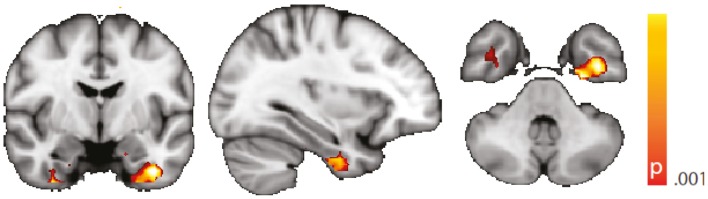
**Spatial map (coronal, sagittal, axial slices) of significantly greater variance of the blood oxygen level-dependent signal in patients with temporal lobe epilepsy (pooled right and left) than in healthy controls**. An additional preprocessing step was performed by erasing volumes associated with large head displacements, as well as surrounding volumes. Color bar indicates *p*-value (thresholded at *p* < 0.001 for display, cluster survives family-wise error correction at *p* < 0.05). Left on figure is right in the brain (coronal and axial slices).

**Figure 6:**
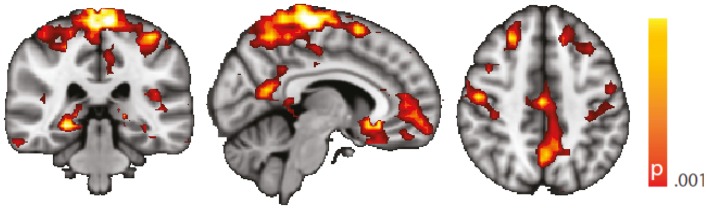
**Spatial map (coronal, sagittal, axial slices) of significantly greater variance of hippocampal dynamic functional connectivity in patients with temporal lobe epilepsy (here: seed in left hippocampus) than in healthy controls**. An additional preprocessing step was performed by erasing volumes associated with large head displacements, as well as six surrounding volumes. Color bar indicates *p*-value (thresholded at *p* < 0.001 for display, cluster survives family-wise error correction at *p* < 0.05). Left on figure is right in the brain (coronal and axial slices).

We are aware of the many factors influencing resting-state – and any other – fMRI studies (51) but not aware of any study formally assessing the order of relevance of the numerous confounding effects. We do know, though, from our own data (12, 31) that sleep alters the neuronal resting-state brain architecture significantly; and motion is known to introduce BOLD signal changes of several magnitudes the size of those commonly induced neuronally (52). In comparison, e.g., effects of sex and age are less pronounced both in terms of extent and intensity, and only optimized analysis methods will reveal such differences (53, 54). Nevertheless, we tried to match both gender and age as much as possible between the examined cohorts. We regressed rigid body motion from the data and in an additional analysis accounting for motion-induced variance in a very conservative way showed that our results were robust w.r.t. motion-induced variance (see “Reproduction of Results Erasing High Amplitude Head Movements” in the Appendix; Figures 5 and 6).

**Figure 7:**
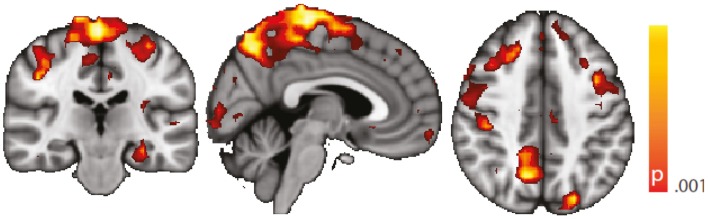
**Spatial map (coronal, sagittal, axial slices) of significantly greater variance of hippocampal dynamic functional connectivity in patients with temporal lobe epilepsy (here: seed in left hippocampus) than in healthy controls**. A sliding window length of 15 s was used for the computation of dynamic functional connectivity time series. Color bar indicates *p*-value (thresholded at *p* < 0.001 for display, cluster survives family-wise error correction at *p* < 0.05). Left on figure is right in the brain (coronal and axial slices).

Regarding confounding fluctuations in wakefulness, to our knowledge, this is the first “wakeful rest” functional connectivity study controlling for and restricting the analysis to true, EEG-defined, awake epochs only and in addition accounting for non-stationarity of the functional connectivity even within the awake state. We advocate both as a desirable standard. To demonstrate independence of our results to the length of the sliding window used in the dynamic functional connectivity analysis, we performed an additional analysis with a different (shorter) window length (see “Robustness against Changes in Sliding Window Length” in the Appendix, Figure 7).

We did not make a strong point about left TLE vs. right TLE comparisons, because we did not observe significant group differences. Evidently, absence of proof is not proof of absence but may be due to patient heterogeneity. Such is almost inevitable in patient studies like this one caused by a variety of factors, such as the range of dyscognitive seizure semiology (not any two patients have the same seizures), seizure frequency, and the potential occurrence of additional generalized tonic clonic seizures, type and location – or by current radiological standards even absence – of structural pathology visible on MRI, IQ, duration of epilepsy, and medication – just to name a few. Although epilepsy syndrome diagnosis relied on multidisciplinary experts reviewing extensive electro-clinical information, we cannot rule out classification errors. But differences within the TLE cohort between left and right TLE patients will not affect our main positive findings, i.e., the group differences between TLE and healthy controls. However, we note that any medication taken by the patients if systematically leading to, e.g., alterations of consciousness could have biased our results; but there is no evidence to date supporting this possibility for the specific patterns we observed.

In addition to these mere technical issues, like in any resting-state study, result interpretation conceptionally is limited given the absence of a task. We based the justification of the study design on the fact that interictal epileptic activity occurs spontaneously at rest as indicated by neuronal discharges measurable with EEG, although we assumed a more general “epileptic process” based on the well-established general clinical observation of interictal cognitive compromitation in TLE. In an attempt to tie our interpretation of our observations more closely to the results, we regressed IQ against BOLD signal variance and dynamic functional connectivity. We report this analysis only in “Correlations Between BOLD Signal Variance/Variance of Dynamic Connectivity Time Series with the Left Hippocampus and VIQ/PIQ” in the Appendix as it needs to be considered of anecdotal character owing to a lack of statistical significance. Causes of the latter include those discussed above.

## Conclusion

We found evidence in support of our hypothesis that, interictally, brain activity is altered in syndrome-specific regions. Assuming that interictal processes like bursts of interictal epileptiform discharges will generate large changes in BOLD amplitude, we analyzed the variance of the BOLD signal and found this increased in anterior temporal regions, which suggest a TLE specific effect. Starting from the anterior temporal lobe, we found hippocampal dynamic connectivity increased in regions, which might explain the hallmark semiological features of complex partial seizures including impaired consciousness (precuneus, frontal cortex), sensory disturbance, and motor automatisms (sensorimotor cortices, supplementary motor area). Taking into account state of the art knowledge about the non-stationarity and state-dependence of functional connectivity, we sought to increase the sensitivity and specificity of our results. More generally, this work encourages the further development of connectivity-derived measures as potential functional imaging biomarkers in TLE.

## Conflict of Interest Statement

The authors declare that the research was conducted in the absence of any commercial or financial relationships that could be construed as a potential conflict of interest.

## Figures and Tables

**Figure A1:**
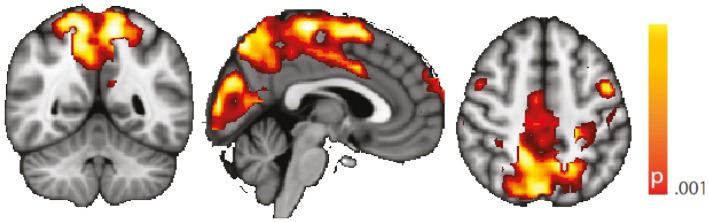
**Spatial map (coronal, sagittal, axial slices) of significantly decreased hippocampal signal linear correlation in patients with temporal lobe epilepsy (here: seed in left hippocampus) than in healthy controls**. Color bar indicates *p*-value (thresholded at *p* < 0.001 for display, cluster survives family-wise error correction at *p* < 0.05). Left on figure is right in the brain (coronal and axial slices).

**Figure A2:**
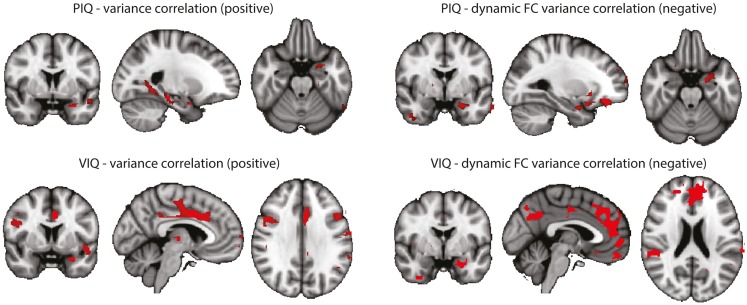
**Correlations between BOLD signal variance/variance of dynamic connectivity time series with the left hippocampus and VIQ/PIQ scores for the TLE group (left + right)**. Statistical significance was determined at *p* < 0.05 with a cluster threshold of 10 voxels. Maps were masked with the regions where the metrics were significantly different between controls and TLE patients. Left: results for PIQ and VIQ correlation with variance. In all cases positive correlations were found. Right: results for PIQ and VIQ correlation with variance of connectivity time series with the left hippocampus. In all cases negative correlations were found.

## Appendix

### Reproduction of results erasing high amplitude head movements

Following the work of Lemieux et al. and Power et al. (55, 56), we have scanned the time course of estimated head movement and erased those volumes associated with a displacement larger than 0.3 mm, as well as the three previous and following volumes. We then reproduced Figures 3 and 4 using this alternative preprocessing. Results are shown in Figure 5 (voxel-wise variance, controls vs. TLE) and Figure 6 (hippocampus-based dynamic functional connectivity variance, controls vs. TLE). It can be appreciated from both figures that the general composition of the patterns discussed in the main text is preserved.

### Robustness against changes in sliding window length

The computation of dynamic functional connectivity time series requires the specification of a sliding window length, which can be seen as a free parameter (even though its correct choice bears an obvious relationship with the time scale where the changes occur). We demonstrate that results are robust against a chance of this parameter by reproducing Figure 4 using a different, shorter window length (15 s). Results are shown in Figure 7. From this figure, it is evident that the main differences between controls and TLE patients still hold using this different window length.

### Standard seed correlation analysis

We performed a standard “static” linear correlation analysis seeded in the left hippocampus. Results of the controls vs. TLE comparison are presented in Figure A1. A decrease in seed connectivity was observed in the patient group relative to the control group in brain areas including visual cortex, precuneus, and sensorimotor cortex. Compared to the regions in which a change in dynamic connectivity variance was observed (Figure 4), these regions overlap but also include a less extended network than that observed in the dynamic analysis; in particular, frontal regions do not appear. To a large extent, for regions having strong baseline connectivity, increased variance of the dynamic connectivity time series implies decreased “static” correlation. The opposite, however, is not true. This points to an origin of the decreased “static” correlation that can be traced to a dynamic transient phenomenon, which we hypothesize corresponds to paroxysmal events.

### Correlations between BOLD signal variance/variance of dynamic connectivity time series with the left hippocampus and VIQ/PIQ

In an attempt to link the results derived from resting-state data more tightly to our interpretation, we performed a correlation analysis of the behavioral measures PIQ/VIQ (obtained close in time to the fMRI experiment) and fMRI signal changes in the regions of increased BOLD signal variance and increased dynamic functional connectivity, respectively (Figure A2). We found positive correlations between the IQ measures and the BOLD signal variance, and negative correlations with the dynamic BOLD functional connectivity. With the caveat of uncorrected significance values – possibly a power or, alternatively, a conceptual problem – this argues for a relationship of our findings obtained at rest with these psychological measures, i.e., a connection between interictal cognitive impairment and the observed changes between the TLE and the control cohorts. That a lower IQ score is linked to higher functional connectivity could be interpreted as paroxysmal interference of the “epileptic process” with normally required and less pronounced variations in functional connectivity. Why at the same time an impaired IQ is associated with reduced variance in the BOLD signal time series in regions characterized by an overall increase in BOLD signal variance in TLE compared to controls – admittedly – is difficult to interpret. Although the functional role of anterior temporal lobe is still debated (57), it might be possible that increased “inertia” (i.e., reduced BOLD signal variance) of the anterior temporal lobe reflects deficits in semantic memory (58).
